# Short-Time Wind Speed Forecast Using Artificial Learning-Based Algorithms

**DOI:** 10.1155/2020/8439719

**Published:** 2020-04-25

**Authors:** Mariam Ibrahim, Ahmad Alsheikh, Qays Al-Hindawi, Sameer Al-Dahidi, Hisham ElMoaqet

**Affiliations:** ^1^Dept. of Mechatronics Eng., Faculty of Applied Technical Sciences, German Jordanian University, Amman 11180, Jordan; ^2^Faculty of Applied Sciences and Industrial Engineering, Deggendorf Institute of Technology, Deggendorf 94469, Germany; ^3^School of Electrical, Information and Media Eng., University of Wuppertal, Wuppertal 42119, Germany; ^4^Dept. of Mechanical & Maintenance Eng., Faculty of Applied Technical Sciences, German Jordanian University, Amman 11180, Jordan

## Abstract

The need for an efficient power source for operating the modern industry has been rapidly increasing in the past years. Therefore, the latest renewable power sources are difficult to be predicted. The generated power is highly dependent on fluctuated factors (such as wind bearing, pressure, wind speed, and humidity of surrounding atmosphere). Thus, accurate forecasting methods are of paramount importance to be developed and employed in practice. In this paper, a case study of a wind harvesting farm is investigated in terms of wind speed collected data. For data like the wind speed that are hard to be predicted, a well built and tested forecasting algorithm must be provided. To accomplish this goal, four neural network-based algorithms: artificial neural network (*ANN*), convolutional neural network (*CNN*), long short-term memory (*LSTM*), and a hybrid model convolutional LSTM (*ConvLSTM*) that combines *LSTM* with *CNN*, and one support vector machine (*SVM*) model are investigated, evaluated, and compared using different statistical and time indicators to assure that the final model meets the goal that is built for. Results show that even though *SVM* delivered the most accurate predictions, *ConvLSTM* was chosen due to its less computational efforts as well as high prediction accuracy.

## 1. Introduction

The need to move towards renewable and clean energy sources has increased considerably over the previous years. Fossil fuels are being misused excessively and eventually will waste away. However, renewable energy (*RE*) sources such as wind, solar, and hydraulic or hydroelectric are regularly replenished and will sustain forever. Grid operators who use *RE* face many challenges which lead to variability and uncertainty in power generation. For instance, in the case of solar power where the existence of clouds that move above solar power plants can narrow power generation for brief intervals of time. Cloud cover may introduce a very quick shift in the outcome of solar structures, but solar energy is still considered to be highly predictable as the sun motion is understood clearly [1]. However, wind power generation is less predictable due to the fact that fluctuations in wind speed are stochastic in nature. This issue will cause a break between supply and demand. So, in order to enhance and optimize renewable wind power generation, wind speed or power production forecasting models are recently being used to resolve this problem. This has led to huge increase in installing wind power plants [2].

As the demand for wind power has increased over the last decades, there is a serious need to set up wind farms and construct facilities depending on accurate wind forecasted data. Collected short-term wind forecasting has a significant effect on the electricity [3], which is also necessary to identify the size of wind farms.

It is obvious that there is a need for an accurate wind forecasting technique to substantially reduce the cost by wind power scheduling [4]. There are several methods which are aimed at short-time wind forecasting (e.g., statistical time series and neural networks). For an advanced and more accurate forecasting, the hybrid models are used. These models combine physical and statistical approaches, short and medium-term models, and combinations of alternative statistical models.

The concept of artificial neural networks (*ANNs*) was first introduced by McCulloch and Pitts [5] in 1943 as a computational model for biological neural networks. Convolutional neural network (*CNN*) was influenced by “Neocognitron” networks which were first introduced by Fukushima in 1980 [6]. *CNN* was based on biological processes which were hierarchical multilayered neural networks used for image processing. These networks are capable of “learning without a teacher” for recognition of various catalyst shapes depending on their geometrical designs [7].

Long short-term memory (*LSTM*) [8] is built upon recurrent neural network (*RNN*) structure. It was designed by Hochreiter and Schmidhuber in 1997. *LSTM* uses the concept proposed in [9] which depends on feedback connections between its layers. Unlike standard feedforward neural networks, *LSTM* can process entire sequences of data (such as voice or video) and not just single data points (such as images).

Support vector machine (*SVM*) [10] is a popular machine learning technique, which is advanced enough to deal with complex data. It is aimed to deal with challenges in classification problems.

In 2016, *Convolutional LSTM (ConvLSTM)* was used to build a video prediction model by Shi et al. [11]. A tool is developed to prognose action-conditioned video that modeled pixel movement, by predicting a distribution over pixel movement from earlier frames. Stacked convolutional *LSTM* was employed to generate motion predictions. This approach has gained the finest outcomes in predicting future object motion.

An end-to-end learning of driving models was developed in [12] using a *LSTM*-based algorithm. A trainable structure for learning how to accurately predict a distribution among upcoming vehicle movement is developed through learning a generic vehicle movement from large-scale crowd-sourced video. The data source used a rapid monocular camera, observations, and past vehicle state. The images were encoded through long short-term memory fully convolutional network (*FCN-LSTM*) to determine the related graphical illustration in every input frame, side by side with a temporal network to use the movement history information. The authors were able to compose an innovative hybrid structure for time-series prediction (*TSP*) that combined an *LSTM* temporal encoder utilizing a fully convolutional visual encoder.

Various papers have been explored in the literature on wind speed forecasting. For instance, a model was introduced by Xu et al. [13] to predict short-term wind speed using *LSTM*, empirical wavelet transformation (*EWT*), and Elman neural network approaches. The *EWT* is implemented to break down the raw wind speed data into multiple sublayers and employ them in Elman neural network (*ENN*) and *LSTM* network to predict the low and high frequency sublayers. Unscented Kalman filter (*UKF*) along with support vector regression (*SVR*) based state-space model was applied by Chen and Yu [14] to efficiently correct the short-term estimation of wind speed chain.

A nonlinear-learning scheme of deep learning time series prediction, *EnsemLSTM*, was developed by Chen et al. [15]. This scheme relied on *LSTM*s, support vector regression machine (*SVRM*), and extremal optimization algorithm (*EO*). Wind speed data are forecasted separately by an array of *LSTM*s that contained covered layers. Neurons are built in every hidden layer. The authors proved that the introduced *EnsemLSTM* is capable of achieving an improved forecasting execution along with the least mean absolute error (MAE), root mean square error (RMSE), mean absolute percentage error (*MAPE*), and the highest *R*-squared (*R*^2^).

A hybrid model constructed of wavelet transform (*WT*) and *SVM* was proposed by Liu et al. [16] to predict wind speed in the short term. The model is improved by genetic algorithm (*GA*), which is implemented to vary essential specifications of *SVM* through reducing the produced errors and searching the optimum specifications to bypass the danger of instability. The presented model is proved to be more efficient than *SVM-GA* model. Wang [17] developed a genetic algorithm of wavelet neural network (*GAWNN*) model. The developed model showed an enhanced operation as compared to the normal wavelet neural network (*WNN*) model in predicting short-term wind power. The model can be located at the beginning of network training as well as in convergent precision.

A prediction model was proposed by Sheikh et al. [18] based on support vector regression (*SVR*) and neural network (*NN*) with backpropagation technique. A windowing data preprocessing was combined with cross and sliding window validations in order to predict wind speed with high accuracy. A hybrid method was presented by Nantian et al. [19], which included variational mode decomposition (*VMD*), partial autocorrelation function (*PACF*) feature selection, and modular weighted regularized extreme learning machine (*WRELM*) prediction. The optimal number of decomposition layers was analyzed by the prediction error of one-step forecasting with different decomposition layers.

A robust forecasting model was proposed by Haijian and Deng [20] by evaluating seasonal features and lag space in wind resource. The proposed model was based on the multilayered perceptron with one hidden layer neural network using the Levenberg–Marquardt optimization method. Least squares support vector machine (*LSSVM*) was used by Xiaodan [21] for the wind speed forecasting. The accuracy of the prediction model parameters was optimized utilizing the particle swarm optimization (*PSO*) to minimize the fitness function in the training process. Ningsih et al. [22] predicted wind speed using recurrent neural networks (*RNNs*) with long short-term memory (*LSTM*). Two optimization models of stochastic gradient descent (*SGD*) and adaptive moment estimation (*Adam*) were evaluated. The Adam method was shown to be better and quicker than SGD with a higher level of accuracy and less deviation from the target.

A nonlinear autoregressive neural network (*NARNET*) model was developed by Datta [23]. The model employed univariate time series data to generate hourly wind speed forecast. The closed loop structure provided error feedback to the hidden layer to generate forecast of the next point. A short-term wind speed forecasting method was proposed by Guanlong et al. [24] using a backpropagation (*BP*) neural network. The weight and threshold values of BP network are trained and optimized by the improved artificial bee colony algorithm. Then, the gathered samples of wind speed are trained and optimized. When training is finished, test samples are used to forecast and validate.

Fuzzy C-means (*FCM*) clustering was used by Gonggui et al. [25] to forecast wind speed. The input data of BP neural network with similar characteristics are divided into corresponding classes. Different BP neural networks are established for each class. The coefficient of variation is used to illustrate the dispersion of data, and statistical knowledge is used to illuminate the input data with large dispersion from the original dataset. Artificial neural networks (*ANNs*) and decision trees (*DTs*) were used by ZhanJie and Mazharul Mujib [26] to analyze meteorological data for the application of data mining techniques through cloud computing in wind speed prediction. The neurons in the hidden layer are enhanced gradually, and the network performance in the form an error is examined.  Table 1 highlights the main characteristics of the existing schemes developed for wind speed forecasting.

The novelty of this work lies in enhancing the accuracy of wind speed forecasting by using a hybrid model called *ConvLSTM* and comparing it with other four commonly used models with optimized lags, hidden neurons, and parameters. This includes testing and comparing the performance of these five different models based on historical data as well employing multi-lags-one-step (*MLOS*) ahead forecasting concept. *MLOS* provided an efficient generalization to new time series data. Thus, it increased the overall prediction accuracy. The remainder of this paper is organized as follows.  Section 2 describes the four learning algorithms in addition to a hybrid algorithm investigated for an accurate wind speed forecasting.  Section 3 illustrates the study methodology.  Section 4 shows a real case study of a wind farm.  Section 5 introduces the results and discussion. Finally, conclusions and future works are presented in Section 6.

### 1.1. Acronyms and Notations

Table 2 illustrates the acronyms and notations used through the paper.

## 2. Prediction Algorithms

In this section, the algorithms used for wind speed forecasting are summarized as follows.

### 2.1. *LSTM* Algorithms

*LSTM* is built in a unique architecture that empowers it to forget the unnecessary information, by turning multiplication into addition and using a function whose second derivative can preserve for a long range before going to zero in order to reduce the vanishing gradient problem (*VGP*). It is constructed of the *sigmoid layer* which takes the inputs *x*_*t*_and *h*_*t*−1_ and then decides by generating the *zeros* which part from the old output should be removed. This process is done through *forget* gate *f*_*t*_. The gate output is given as *f*_*t*_*∗c*_*t*−1_. After that, a vector of all the possible values from the new input is created by tan *h layer*. These two results are multiplied to renew the old memory *c*_*t*−1_ that gives *c*_*t*_. In other words, the *sigmoid layer* decides which portions of the cell state will be the outcome. Then, the outcome of the sigmoid gate is multiplied by all possible values that are set up through tan *h*. Thus, the output consists of only the parts that are decided to be generated.

*LSTM* networks [8] are part of recurrent neural networks (*RNN*s), which are capable of learning long-term dependencies and powerful for modeling long-range dependencies. The main criterion of the *LSTM* network is the memory cell which can memorize the temporal state. It is also shaped by the addition or removal of information through three controlling gates. These gates are the *input* gate, *forget* gate, and *output* gate. *LSTM*s are able to renew and control the information flow in the block using these gates in the following equations:

(1) $$\begin{array}{c} si_{t}=\sigma \left(x_{t}\cdot \theta_{xi}+h_{t-1}\cdot \theta_{hi}+\theta_{i_{\text{bias}}}\right), \end{array}$$

(2) $$\begin{array}{c} f_{t}=\sigma \left(x_{t}\cdot \theta_{xf}+h_{t-1}\cdot \theta_{hf}+\theta_{f_{\text{bias}}}\right), \end{array}$$

(3) $$\begin{array}{c} {\tilde{c}}_{t}=\tan h\left(x_{t}\cdot \theta_{x\tilde{c}}+h_{t-1}\cdot \theta_{h\tilde{c}}+\theta_{{\tilde{c}}_{\text{bias}}}\right), \end{array}$$

(4) $$\begin{array}{c} c={\tilde{c}}_{t}⊙i_{t}+c_{t-1}⊙f_{t}, \end{array}$$

(5) $$\begin{array}{c} o_{t}=\sigma \left(x_{t}\cdot \theta_{xo}+h_{t-1}\cdot \theta_{ho}+\theta_{o_{\text{bias}}}\right), \end{array}$$

(6) $$\begin{array}{c} h_{t}=o_{t}⊙\;\tan h\left(c_{t}\right), \end{array}$$

where “·” presents matrix multiplication, “⊙” is an elementwise multiplication, and “θ” stands for the weights. $\tilde{c}$ is the input to the cell *c* which is gated by the input gate, while *o*_*t*_ is the output. The nonlinear functions *σ* and tan *h* are applied elementwise, where *σ*(*x*)=1/1+*e*^−*x*^. Equations (1) and (2) establish gate activations, equation (3) indicates cell inputs, equation (4) determines the new cell states, where the ‘memories' are stored or deleted, and equation (5) results in the output gate activations which are shown in equation (6), the final output.

### 2.2. *CNN* Algorithms

*CNN* is a feed-forward neural network. To achieve network architecture optimization and solve the unknown parameters in the network, the attributes of a two-dimensional image are excerpted and the backpropagation algorithms are implemented. To achieve the final outcome, the sampled data are fed inside the network to extract the needed attributes within prerefining. Next, the classification or regression is applied [27].

The *CNN* is composed of basically two types of layers: the convolutional and the pooling layers. The neurons are locally connected within the convolution layer and the preceding layer. Meanwhile, the neurons' local attributes are obtained. The local sensitivity is found through the pooling layer to obtain the attributes repeatedly. The existence of the convolution and the pooling layers minimizes the attribute resolution and the number of network specifications which require enhancement.

*CNN* typically describes data and constructs them as a two-dimensional array and is extensively utilized in the area of image processing. In this paper, *CNN* algorithm is configured to predict the wind speed and fit it to process a one-dimensional array of data. In the preprocessing phase, the one-dimensional data are reconstructed into a two-dimensional array. This enables *CNN* machine algorithm to smoothly deal with data. This creates two files: the property and the response files. These files are delivered as inputs to *CNN*. The response file also contains the data of the expected output value.

Each sample is represented by a line from the property and the response files. Weights and biases can be obtained as soon as an acceptable number of samples to train the *CNN* is delivered. The training continues by comparing the regression results with the response values in order to reach the minimum possible error. This delivers the final trained *CNN* model, which is utilized to achieve the needed predictions.

The fitting mechanism of *CNN* is pooling. Various computational approaches have proved that two approaches of pooling can be used: the average pooling and the maximum pooling. Images are stationary, and all parts of image share similar attributes. Therefore, the pooling approach applies similar average or maximum calculations for every part of the high-resolution images. The pooling process leads to reduction in the statistics dimensions and increase in the generalization strength of the model. The results are well optimized and can have a lower possibility of over fitting.

### 2.3. *ANN* Algorithms

*ANN* has three layers which build up the network. These are input, hidden, and output layers. These layers have the ability to correlate an input vector to an output scalar or vector using activation function in various neurons. The *j*_*th*_ hidden neuron *Z*_*j*_can be computed by the *p* inputs and *m* hidden neurons using the following equation [14]:

(7) $$\begin{array}{c} Z_{j}=f_{h}\left(\overset{p}{\underset{i=1}{\sum }}w_{ij}y_{k-i}\right), \end{array}$$

where *w*_*ij*_ is the connection weight from the *i*_*th*_ input node to the *j*_*th*_ hidden node, *y*_*k*−*i*_ is *i*-step behind previous wind speed, and *f*_*h*_(.) is the activation function in the hidden layer. Therefore, the future wind speed can be predicted through

(8) $$\begin{array}{c} {\hat{y}}_{k}=f_{o}\left(\overset{m}{\underset{j=1}{\sum }}w_{j}z_{j}\right), \end{array}$$

where *w*_*j*_ is the connection weight from the *j*_*th*_ hidden node to the output node and ${\hat{y}}_{k}\;$ is the predicted wind speed at the *k*_*th*_ sampling moment while *f*_0_ is the activation function for the output layer. By minimizing the error between the actual and the predicted wind speeds, *y*_*k*_ and ${\hat{y}}_{k}$, respectively, using Levenberg–Marquardt (*LM*) algorithm, the nonlinear mapping efficiency of *ANN* can be obtained [28].

### 2.4. *SVM* Algorithms

Assuming a set of samples {*x*_*i*_, *y*_*i*_}, where *i*=1,  2,…,  *N*, with input vector *x*_*i*_  ∈  *R*_*m*_ and output vector *y*_*i*_  ∈  *R*_*m*_. The regression obstacles aim to identify a function *f*_(*x*)_that describes the correlation between inputs and outputs. The interest of *SVR* is to obtain a linear regression in the high-dimensional feature space delivered by mapping the primary input set utilizing a preknown function *ϕ*(*x*(.))and to minimize the structure risk *R*[*f*]. This mechanism can be written as follows [15]:

(9) $$\begin{array}{c} \;f\left(x\right)=w^{T}\phi_{\left(x\right)}+b,\\ \;R\left[f\right]=\frac{1}{2}{\left\|W\right\|}^{2}+C\overset{N}{\underset{i=1}{\sum }}L\left(x_{i}y_{i}f_{\left(x_{i}\right)}\right), \end{array}$$

where *W*, *b*, and *C*, respectively, are the regression coefficient vector, bias term, and punishment coefficient. *L*(*x*_*i*_, *y*_*i*_, *f*_(*x*_*i*_)_) is the *e*-insensitive loss function. The regression problem can be handled by the following constrained optimization problem:

(10) $$\begin{array}{c} \begin{array}{cc} \min, & \frac{1}{2}{\left\|W\right\|}^{2}+C\overset{N}{\underset{i=1}{\sum }}L\left(\zeta_{i}\zeta_{i}^{*}\right),\\ \text{s}.\text{t.}, & y_{i}-\left(w^{T}\phi_{\left(x\right)}+b\right)\;\leq \;\varepsilon +\;\zeta_{i}\;\left(w^{T}\phi_{\left(x\right)}+b\right)-y_{i}\;\leq \;\varepsilon +\;\zeta_{i}^{*}\zeta_{i},\zeta_{i}^{*}\geq 0,\;\;i=1,\;2,\ldots ,\;N, \end{array} \end{array}$$

where *ζ*_*i*_and *ζ*_*i*_^*∗*^represent the slack variables that let constraints feasible. By using the Lagrange multipliers, the regression function can be written as follows:

(11) $$\begin{array}{c} f\left(x\right)=\overset{N}{\underset{i=1}{\sum }}\left(a_{i}-a_{i}^{*}\right)K\left(x_{i}x_{j}\right)+b, \end{array}$$

where *a*_*i*_and *a*_*i*_^*∗*^are the Lagrange multipliers that fulfil the conditions *a*_*i*_ ≥ 0,  *a*_*i*_^*∗*^ ≥ 0 and ∑_*i*=1_^*N*^(*a*_*i*_ − *a*_*i*_^*∗*^)=0.*K*(*x*_*i*_*x*_*j*_) is a general kernel function. In this study, the well-known radial basis function (*RBF*) is chosen here as the kernel function:

(12) $$\begin{array}{c} \;K\left(x_{i}x_{j}\right)=\exp\left(-\frac{{\left\|x_{i}-x_{j}\right\|}^{2}}{2\sigma^{2}}\right), \end{array}$$

where *σ* defines the *RBF* kernel width [15].

### 2.5. *ConvLSTM* Algorithm

*ConvLSTM* is designed to be trained on spatial information in the dataset, and its aim is to deal with 3-dimentional data as an input. Furthermore, it exchanges matrix multiplication through convolution operation on every *LSTM* cell's gate. By doing so, it has the ability to put the underlying spatial features in multidimensional data. The formulas that are used at each one of the gates (input, forget, and output) are as follows:

(13) $$\begin{array}{c} i_{t}=\sigma \left(W_{xi}*x_{t}+W_{hi}*h_{t-1}+b_{i}\right)\;,\\ f_{t}=\sigma \left(W_{xf}*x_{t}+W_{hf}*h_{t-1}+b_{f}\right),\\ o_{t}=\sigma \left(W_{xo}*x_{t}+W_{ho}*h_{t-1}+b_{o}\right),\\ C_{t}=f_{t}\circ C_{t-1}\tan h\left(W_{xc}*x_{t}+W_{hc}*h_{t-1}+b_{c}\right),\\ H_{t}=o-t\circ \;\tan h\left(c_{t}\right), \end{array}$$

where *i*_*t*_, *f*_*t*_, and *o*_*t*_ are input, forget, and output gates and *W* is the weight matrix, while *x*_*t*_  is the current input data. *h*_*t*−1_ is the previous hidden output, and *C*_*t*_ is the cell state.

The difference between these equations in *LSTM* is that the matrix multiplication (·) is substituted by the convolution operation (^*∗*^) between *W* and each *x*_*t*_, *h*_*t*−1_ at every gate. By doing so, the whole connected layer is replaced by a convolutional layer. Thus, the number of weight parameters in the model can be significantly reduced.

## 3. Methodology

Due to the nonlinear, nonstationary attributes and the stochastic variations in the wind speed time series, the accurate prediction of wind speed is known to be a challenging effort [29]. In this work, to improve the accuracy of the wind speed forecasting model, a comparison between five models is conducted to forecast wind speed considering available historical data. A new concept called multi-lags-one-step (*MLOS*) ahead forecasting is employed to illustrate the effect on the five models accuracies. Assume that we are at time index  *X*_*t*_. To forecast one output element in the future *X*_*t*+1_, the input dataset can be splitted into many lags (past data) *X*_*t*−*I*_, where I ∈ {1–10}. By doing so, the model can be trained on more elements before predicting a single event in the future. In addition to that, the model accuracy showed an improvement until it reached the optimum lagging point, which had the best accuracy. Beyond this point, the model accuracy is degraded as it will be illustrated in the Results section.

**Figure 1:**
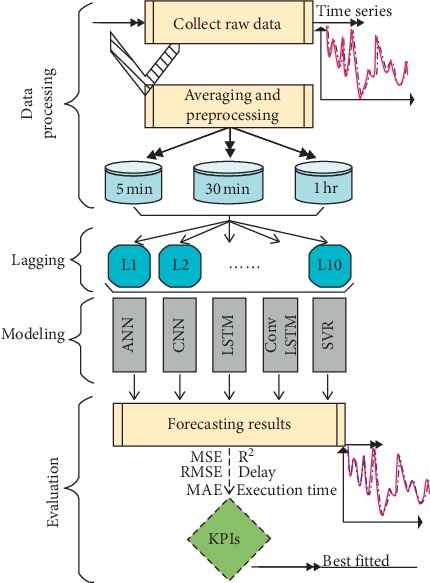
The proposed forecasting methodology.

Figure 1 illustrates the workflow of the forecasting model. Specifically, the proposed methodology entails four steps.

In Step 1, data have been collected and averaged from 5 minutes to 30 minutes and to 1 hour, respectively. The datasets are then standardized to generate a mean value of 0 and standard deviation of 1. The lagging stage is very important in Step 2, as the data are split into different lags to study the effect of training the models on more than one element (input) to predict a single event in the future. In Step 3, the models have been applied taking into consideration that some models such as *CNN*, *LSTM*, and *ConvLSTM* need to be adjusted from matrix shape perspective. These models normally work with 2D or more. In this stage, manipulation and reshaping of matrix are conducted. For the sake of checking and evaluating the proposed models, in Step 4, three main metrics are used to validate the case study (MAE, RMSE, and *R*^2^). In addition, the execution time and optimum lag are taken into account to select the best model.

Algorithm 1 illustrates the training procedure for *ConvLSTM*.

## 4. Collected Data

Table 3 illustrates the characteristics of the collected data in 5 minutes time span. The data are collected from a real wind speed dataset over a three-year period from the West Texas Mesonet, with 5-minute observation period from near Lake Alan, Garza [30]. The data are processed through averaging from 5 minutes to 30 minutes (whose statistical characteristics are given in Table 4) and one more time to 1 hour (whose statistical characteristics are also given in Table 5). The goal of averaging is to study the effect of reducing the data size in order to compare the five models and then select the one that can achieve the highest accuracy for the three dataset cases. As shown in the three tables, the data sets are almost identical and reserved with their seasonality. Also, they are not affected by the averaging process.

The data have been split into three sets (training, validation, and test) with fractions of 53 : 14 : 33.

## 5. Results and Discussion

To quantitatively evaluate the performance of the predictive models, four commonly used statistical measures are tested [20]. All of them measure the deflection between the actual and predicted wind speed values. Specifically, RMSE, MAE, and *R*^2^ are as follows:

(14) $$\begin{array}{c} \text{RMSE}=\sqrt{\frac{\sum_{i=1}^{N_{i}}{\left(y_{i}-{\hat{y}}_{i}\right)}^{2}}{N_{i}}},\\ \text{ MAE}=\frac{1}{N}\sum_{i=1}^{N}\left|y_{i}-{\hat{y}}_{i}\right|,\\ R^{2}=1-\frac{\sum_{i=1}^{N_{i}}{\left(y_{i}-{\hat{y}}_{i}\right)}^{2}}{\sum_{i=1}^{N_{i}}{\left(y_{i}-{\bar{y}}_{i}\right)}^{2}}, \end{array}$$

where *y*_*i*_ and ${\hat{y}}_{i}$ are the actual and the predicted wind speed, respectively, while ${\bar{y}}_{i}$ is the mean value of actual wind speed sequence. Typically, the smaller amplitudes of these measures indicate an improved forecasting procedure, while *R*^2^ is the goodness-of-fit measure for the model. Therefore, the larger its value is, the fitter the model will be. The testbed environment configuration is as follows:

1. CPU : Intel (R) Core(TM) i7-8550U CPU @ 1.80 GHz, 2001 Mhz, 4 Core (s), 8 Logical Processor (s)
2. RAM : Installed Physical Memory 16.0 GB
3. GPU : AMD Radeon(TM) RX 550 10 GB
4. Framework: Anaconda 2019.07, Python 3.7

Table 6 illustrates the chosen optimized internal parameters (hyperparameters) for the forecasting methods used in this work. For each method, the optimal number of hidden neurons is chosen to achieve the maximum *R*^2^ and the minimum RMSEand MAE values.

After the implementation of *CNN*, *ANN*, *LSTM*, *ConvLSTM*, and *SVM,* it was noticed that the most fitted model was chosen depending on its accuracy in predicting future wind speed values. Thus, the seasonality is considered for the forecast mechanism. The chosen model has to deliver the most fitted data with the least amount of error, taking into consideration the nature of the data and not applying naive forecasting on it.

**Figure 2:**
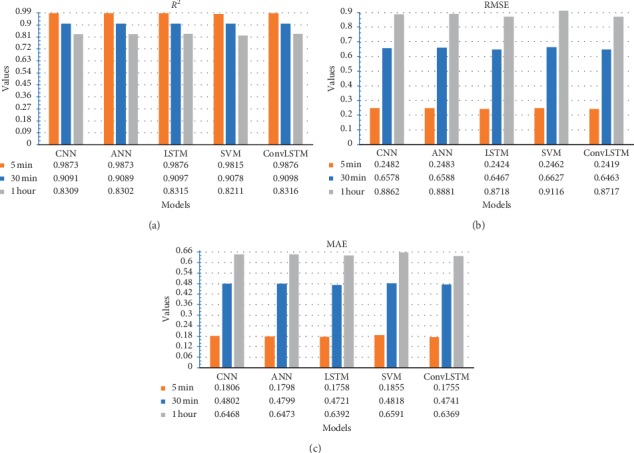
Models' key performance indicators (KPIs).

To achieve this goal, the statistical error indicators are calculated for every model and time lapse and fully represented as Figure 2 illustrates. The provided results suggest that the *ConvLSTM* model has the best performance as compared to the other four models. The chosen model has to reach the minimum values of RMSE and MAE *while* maximum *R*^2^ value.

**Figure 3:**
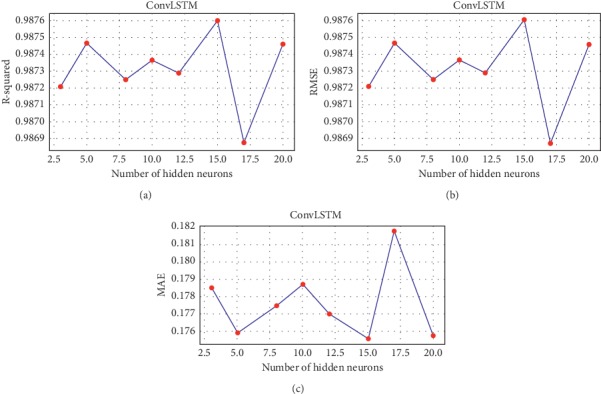
*ConvLSTM* measured statistical values and number of hidden neurons.

**Figure 4:**
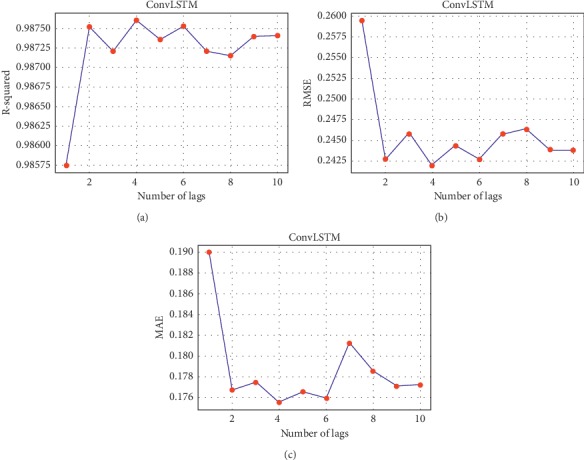
*ConvLSTM* measured statistical values and number of lags.

Different parameters are also tested to ensure the right decision of choosing the best fitted model. The optimum number of lags which is presented in Table 7 is one of the most important indicators in selecting the best fitted model. Since the less historical points are needed by the model, the computational effort will be less as well. For each method, the optimal number of lags is chosen to achieve the maximum *R*^2^and the minimum RMSE and MAE values. For instance, Figures 3 and 4 show the relation between the statistical measures and the number of lags and hidden neurons, respectively, for the proposed *ConvLSTM* method for the 5 minutes time span case. It can be seen that 4 lags and 15 hidden neurons achieved maximum *R*^2^ and minimum RMSE and MAE values.

The execution time shown in Table 8 is calculated for each method and time lapse to assure that the final and chosen model is efficient and can effectively predict future wind speed. The shorter the time for execution is, the more efficient and helpful the model will be. This is also a sign that the model is efficient for further modifications. According to Table 8, the *ConvLSTM* model beats all other models in the time that it needed to process the historical data and deliver a final prediction; *SVM* needed 54 minutes to accomplish the training and produce testing results while *ConvLSTM* made it in just 1.7 minutes. This huge difference between them has made the choice of using *ConvLSTM*.

**Figure 5:**
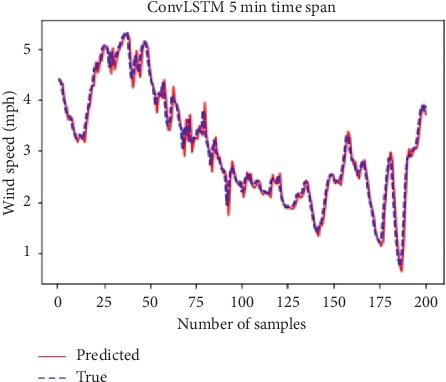
*ConvLSTM* true/predicted wind speed and number of samples.

Figure 5 shows that the 5-minute lapse dataset is the most fitted dataset to our chosen model. It declares how accurate the prediction of future wind speed will be.

**Figure 6:**
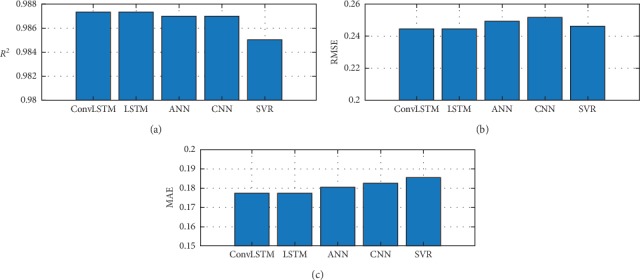
Average performance metrics obtained on the test dataset using 50 cross validation procedure.

For completeness, to effectively evaluate the investigated forecasting techniques in terms of their prediction accuracies, 50 cross validation procedure is carried out in which the investigated techniques are built and then evaluated on 50 different training and test datasets, respectively, randomly sampled from the available overall dataset. The ultimate performance metrics are then reported as the average and the standard deviation values of the 50 metrics obtained in each cross validation trial. In this regard, Figure 6 shows the average performance metrics on the test dataset using the 50 cross validation procedure. It can be easily recognized that the forecasting models that employ the *LSTM* technique outperform the other investigated techniques in terms of the three performance metrics, *R*^2^, RMSE, and MAE.

From the experimental results of short-term wind speed forecasting shown in Figure 6, we can observe that *ConvLSTM* performs the best in terms of forecasting metrics (*R*^2^, RMSE, and MAE) as compared to the other models (i.e., *CNN*, *ANN*, *SVR*, and *LSTM*). The related statistical tests in Tables 6 and 7, respectively, have proved the effectiveness of *ConvLSTM* and its capability of handling noisy large data. *ConvLSTM* showed that it can produce high accurate wind speed prediction with less lags and hidden neurons. This was indeed reflected in the results shown in Table 8 with less computation time as compared to the other tested models. Furthermore, we introduced the multi-lags-one-step (*MLOS*) ahead forecasting combined with the hybrid *ConvLSTM* model to provide an efficient generalization for new time series data to predict wind speed accurately. Results showed that *ConvLSTM* proposed in this paper is an effective and promising model for wind speed forecasting.

Similar to our work, the proposed *EnsemLSTM* model by Chen et al. [15] contained different clusters of *LSTM* with different hidden layers and hidden neurons. They combined *LSTM* clusters with *SVR* and external optimizer in order to enhance the generalization capability and robustness of their model. However, their model showed a high computational complexity with mediocre performance indices. Our proposed *ConvLSTM* with *MLOS* assured boosting the generalization and robustness for the new time series data as well as producing high performance indices.

## 6. Conclusions

In this study, we proposed a hybrid deep learning-based framework *ConvLSTM* for short-term prediction of the wind speed time series measurements. The proposed dynamic prediction model was optimized for the number of input lags and the number of internal hidden neurons. Multi-lags-one-step (*MLOS*) ahead wind speed forecasting using the proposed approach showed superior results compared to four other different models built using standard *ANN*, *CNN*, *LSTM*, and *SVM* approaches. The proposed modeling framework combines the benefits of *CNN* and *LSTM* networks in a hybrid modeling scheme that shows highly accurate wind speed prediction results with less lags and hidden neurons, as well as less computational complexity. For future work, further investigation can be done to improve the accuracy of the *ConvLSTM* model, for instance, increasing and optimizing the number of hidden layers, applying a multi-lags-multi-steps (*MLMS*) ahead forecasting, and introducing reinforcement learning agent to optimize the parameters as compared to other optimization methods.

## Figures and Tables

**Algorithm 1:**
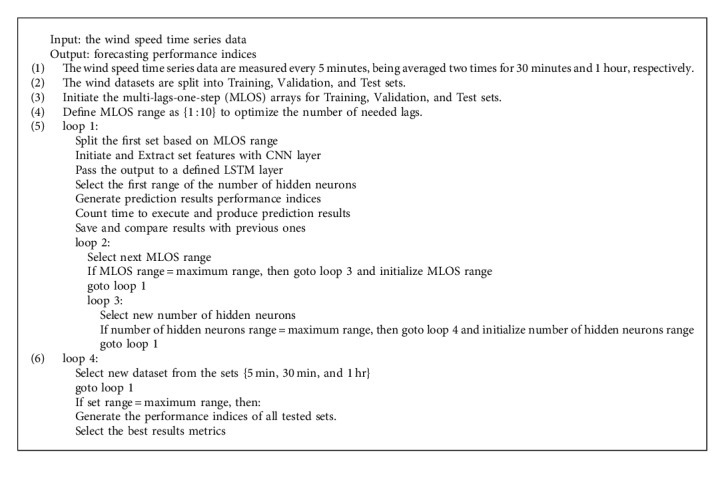
* ConvLSTM* training.

**Table 1:** Main characteristics of the existing wind speed forecasting schemes.

Aim	Technique	Merits/outcomes	Demerits	Dataset
Hybrid wind speed prediction [13]	Empirical wavelet transformation (*EWT*), long short-term memory neural network, and a deep learning algorithm.	The proposed model has the satisfactory multistep forecasting results.	The performance of the EWT for the wind speed multistep forecasting has not been studied	Four sets of original wind speed series including 700 samples.

Wind speed forecasting [14]	Unscented Kalman filter (*UKF*) is integrated with support vector regression (*SVR*) model	The proposed method has better performance in both one-step-ahead and multistep-ahead predictions than *ANNs, SVR*, autoregressive, and autoregressive integrated with Kalman filter models	Needs to develop the predictive model-based control and optimization strategies for wind farm operation.	Center for Energy Efficiency and Renewable Energy at University of Massachusetts

Wind speed forecasting [15]	Long short-term memory neural networks, support vector regression machine, and extremal optimization algorithm.	The proposed model can achieve a better forecasting performance than *ARIMA, SVR, ANN, KNN*, and *GBRT* models.	Needs to consider more interrelated features like weather conditions, human factors, and power system status.	A wind farm in Inner Mongolia, China

A hybrid short-term wind speed forecasting [16]	Wavelet transform (*WT*), genetic algorithm (*GA*), and support vector machines (*SVMs)*	The proposed method is more efficient than a persistent model and a *SVM-GA* model without *WT*	Needs to augment external information such as the air pressure, precipitation, and air humidity besides the temperature.	The wind speed data every 0.5 h in a wind farm of North China in September 2012

Short-term wind speed prediction [18].	Support vector regression (*SVR*) and artificial neural network (*ANN*) with backpropagation	The proposed *SVR* and *ANN* models are able to predict wind speed with more than 99% accuracy.	Computationally expensive	Historical dataset (2008–2014) of wind speed of Chittagong costal area from Bangladesh Meteorological Division (*BMD*)

Hybrid wind speed forecasting [19]	Variational mode decomposition (*VMD*), the partial autocorrelation function (*PACF*), and weighted regularized extreme learning machine (*WRELM*)	(i) The *VMD* reduces the influences of randomness and volatility of wind speed. (ii) *PACF* reduces the feature dimension and complexity of the model. (iii) *ELM* improves the prediction accuracy.	The forecasting accuracy of two-step-ahead and three-step-ahead predictions declined to different degrees.	USA National Renewable Energy Laboratory (*NREL*) in 2004.

Short-term wind speed forecasting [20].	Wavelet analysis and AdaBoosting neural network.	(i) Benefits the analysis of the wind speed's randomness and optimal neural network's structure. (ii) It can be used to promote the model's configuration and show the confidence in high-accuracy forecasting.	Needs to consider the dynamical model with ability of error correction and adaptive adjustment.	USA National Renewable Energy Laboratory (*NREL*) in 2004.

Short-term wind speed forecasting [21].	Support vector machine (*SVM*) with particle swarm optimization (*PSO*)	The proposed model has the best forecasting accuracy compared to classical *SVM* and backpropagation neural network models.	Needs to consider additional information for efficient forecasting such as season and weather variables.	Wind farm data in China in 2011.

Wind speed predictions [22].	Recurrent neural network (*RNN*) with long short-term memory (*LSTM*).	The model provides 92.7% accuracy for training data and 91.6% for new data.	High rate epochs increased the process time and eventually provided low accuracy performance.	Nganjuk Meteorology and Geophysics Agency (*BMKG*), East Java (2008–2017).

Forecasting multistep-ahead wind speed [23]	*NARNET* model to forecast hourly wind speed using an artificial neural network (*ANN*).	The model is cost effective and can work with minimum availability of statistical data	(i) Faulty measurements of inputs are likely to affect the model parameters. (ii) Removing rapid changes using a low-pass filter might result in neglecting important information.	Meteorological data from the National Oceanic and Atmospheric Administration (*NOAA*) located in Dodge City, Kansas (January 2010 -December 2010).

Short-term wind speed prediction [24]	Backpropagation (*BP*) neural network based on improved artificial bee colony algorithm (*ABC-BP*).	The model has high precision and fast convergence rate compared with traditional and genetic *BP* neural networks.	Sensitive for noisy data. Therefore, data should be filtered, which may affect the nature of data.	Wind farm in Tianjin, China (December 2013–January 2014).

Short-term wind speed forecasting [25] 2019	Fuzzy C-means clustering (*FCM*) and improved mind evolutionary algorithm-BP (*IMEA-BP*).	The proposed model is suitable for one-step forecasting and enhances the accuracy of multistep forecasting.	The accuracy of multistep forecasting needs to be further improved.	Wind farm in China

Predicting wind speed [26].	Artificial neural network and decision tree algorithms	The platform has the ability of mass storage of meteorological data, and efficient query and analysis of weather forecasting.	Needs improvement in order to forecast more realistic weather parameters.	Meteorological data provided by the Dalian Meteorological Bureau (2011–2015)

Our scheme	Employing multi-lags-one-step (*MLOS*) ahead forecasting technique with artificial learning-based algorithms	The provided results suggest that the *ConvLSTM* model has the best performance as compared to *ANN, CNN, LSTM*, and *SVM* models.	Increasing the number of hidden layers may increase the computational time exponentially.	National Wind Institution, West Texas Mesonet (2012–2015)

**Table 2:** Acronyms and notations used.

Category	Items/symbols	Description
Acronyms	*ANN*	Artificial neural network
	*CNN*	Convolutional neural network
	*LSTM*	Long short-term memory
	*ConvLSTM*	Convolutional LSTM hybrid model
	*SVM*	Support vector machine
	*RE*	Renewable energy
	*RNN*	Recurrent neural network
	*EWT*	Empirical wavelet transformation
	*ENN*	Elman neural network
	*FC-LSTM*	Fully connected-long short-term memory
	*FCN-LSTM*	Long short-term memory fully convolutional network
	*TSP*	Time-series prediction
	*UKF*	Unscented Kalman filter
	*SVR*	Support vector regression
	*SVRM*	Support vector regression machine
	*EO*	Extremal optimization
	MAE	Mean absolute error
	RMSE	Root mean square error
	*MAPE*	Mean absolute percentage error
	*R* ^2^	*R*-squared
	*WT*	Wavelet transform
	*GA*	Genetic algorithm
	*GAWNN*	Genetic algorithm of wavelet neural network
	*WNN*	Wavelet neural network
	*MLOS*	Multi-lags-one-step
	*VGP*	Vanishing gradient problem
	*LM*	Levenberg–Marquardt
	*RBF*	Radial basis function
Notations	*f*_*t*_	Forget gate
	*C*_*t*_	The cell state
	*si*_*t*_	Input gate
	*x*_*t*_	Current input data
	*h*_*t*−1_	The previous hidden output
	${\tilde{c}}_{t}$	Input to cell c
	*c*	Memory cell
	${\tilde{c}}_{t}$	Input to cell c
	*i*_*t*_	Input gate
	*C*_*t*−1_	Past cell status
	*O*_*t*_	Output gate
	*h*_*t*_	Hidden state
	·	Matrix multiplication
	⊙	An elementwise multiplication
	*θ*	Weight
	$\tilde{c}$	The input to the cell
	*σ*	Nonlinear function
	*z*_*j*_	The *j*_th_ hidden neuron
	*p*	Number of inputs to the network
	*m*	Number of hidden neurons
	*w*_*ij*_	The connection weight from the *i*_th_ input node to the *j*_th_ hidden node
	*y*_*k*−*i*_	*i*-step behind previous wind speed
	*f*_*h*_(.)	The activation function in the hidden layer
	*w*_*j*_	The connection weight from the *j*_th_ hidden node to the output node
	${\hat{y}}_{k}$	The predicted wind speed at the *k*_th_ sampling moment
	*f*_*o*_	The activation function for the output layer
	*y*_*k*_	Actual wind speed
	*x*_*i*_	Input vector
	*y*_*i*_	Output vector
	*R*_*m*_	Regularized function
	*f*(*x*)	A function that describes the correlation between inputs and outputs.
	*ϕx*(.)	Preknown function
	*R*[*f*]	Structure risk
	*w*	The regression coefficient vector
	*b*	Bias term
	*c*	Punishment coefficient
	*L*(*x*_*i*_, *y*_*i*_, *f*_(*x*_*i*_)_)	The *ε*-insensitive loss function
	*ε*	Threshold
	*ζ*_*i*_, *ζ*_*i*_^*∗*^	Slack variables that let constraints feasible
	*a*_*i*_, *a*_*i*_^*∗*^	The Lagrange multipliers
	*K*(*x*_*i*_*x*_*j*_)	The kernel function
	*W*	The weight matrix
	^*∗*^	Convolution operation
	*b*_*i*_, *b*_*f*_, *b*_*c*_	Bias vectors
	∘	Hadamard product
	*H*_*t*_	Hidden state
	*X*_*t*_	Current wind speed measure
	*X*_*t*−1_	Previous wind speed measure
	*X*_*t*+1_	Future wind speed measure

**Table 3:** Dataset characteristic for 5 min sample.

Dataset	Max	Median	Min	Mean	Std
All datasets	18.73	3.53	0.01	3.91	2.10
Training dataset	18.73	3.47	0.01	3.83	2.05
Test dataset	14.87	3.67	0.01	4.05	2.20

**Table 4:** Dataset characteristic for 30 min sample.

Dataset	Max	Median	Min	Mean	Std
All datasets	17.66	3.53	0.01	3.91	2.08
Training dataset	17.66	3.47	0.01	3.837309	2.02
Test dataset	14.32	3.67	0.02	4.05	2.18

**Table 5:** Dataset characteristic for 1 hour sample.

Dataset	Max	Median	Min	Mean	Std
All datasets	17.61	3.53	0.07	3.91	2.05
Training dataset	17.61	3.46	0.07	3.83	2.00
Test dataset	14.22	3.66	0.07	4.05	2.15

**Table 6:** Optimized internal parameters for the forecasting methods.

*Method*	Sets of parameters
*LSTM*	5 min: 17 hidden neurons
	30 min: 20 hidden neurons
	1 hour: 8 hidden neurons

*CNN*	5 min: 15 hidden neurons
	30 min: 5 hidden neurons
	1 hour: 15 hidden neurons

*ConvLSTM*	5 min: 15 hidden neurons
	30 min: 8 hidden neurons
	1 hour: 20 hidden neurons

*ANN*	5 min: 15 hidden neurons
	30 min: 15 hidden neurons
	1 hour: 20 hidden neurons

*SVR*	5 min: *C* = 7, *ε* = 0.1, *γ* = 0.2.
	30 min: *C* = 1, *ε* = 0.25, *γ* = 0.15.
	1 hour: *C* = 1, *ε* = 0.1, *γ* = 0.05.

**Table 7:** Optimum number of lags.

*Method*	5 min	30* *min	1 hour
*CNN*	9	4	4
*ANN*	3	3	3
*LSTM*	7	6	10
*ConvLSTM*	4	5	8
*SVR*	9	4	5

**Table 8:** Execution time.

	(5 min) time (min)	(30* *min) time (min)	(1 hour) time (min)
*ConvLSTM*	1.7338	0.3849	0.1451
*SVR*	54.1424	0.8214	0.2250
*CNN*	0.87828	0.1322	0.0708
*ANN*	0.7431	0.2591	0.0587
*LSTM*	1.6570	0.3290	0.1473

## Data Availability

The wind speed data used in this study have been taken from the West Texas Mesonet of the US National Wind Institute (http://www.depts.ttu.edu/nwi/research/facilities/wtm/index.php). Data are provided freely for academic research purposes only and cannot be shared/distributed beyond academic research use without permission from the West Texas Mesonet.
